# Beyond the Human Genome Project: The Age of Complete Human Genome Sequences and Pangenome References

**DOI:** 10.1146/annurev-genom-021623-081639

**Published:** 2024-08-06

**Authors:** Dylan J. Taylor, Jordan M. Eizenga, Qiuhui Li, Arun Das, Katharine M. Jenike, Eimear E. Kenny, Karen H. Miga, Jean Monlong, Rajiv C. McCoy, Benedict Paten, Michael C. Schatz

**Affiliations:** 1 Department of Biology, Johns Hopkins University, Baltimore, Maryland, USA;; 2 Genomics Institute, University of California, Santa Cruz, California, USA;; 3 Department of Computer Science, Johns Hopkins University, Baltimore, Maryland, USA;; 4 Department of Genetic Medicine, Johns Hopkins University School of Medicine, Baltimore, Maryland, USA;; 5 Institute for Genomic Health, Icahn School of Medicine at Mount Sinai, New York, NY, USA;; 6 Department of Biomolecular Engineering, University of California, Santa Cruz, California, USA; 7 Institut de Recherche en Santé Digestive, Université de Toulouse, INSERM, INRA, ENVT, UPS, Toulouse, France;

**Keywords:** telomere-to-telomere, pangenome, reference genome sequence, genetic diversity, precision medicine

## Abstract

The Human Genome Project was an enormous accomplishment, providing a foundation for countless explorations into the genetics and genomics of the human species. Yet for many years, the human genome reference sequence remained incomplete and lacked representation of human genetic diversity. Recently, two major advances have emerged to address these shortcomings: complete gap-free human genome sequences, such as the one developed by the Telomere-to-Telomere Consortium, and high-quality pangenomes, such as the one developed by the Human Pangenome Reference Consortium. Facilitated by advances in long-read DNA sequencing and genome assembly algorithms, complete human genome sequences resolve regions that have been historically difficult to sequence, including centromeres, telomeres, and segmental duplications. In parallel, pangenomes capture the extensive genetic diversity across populations worldwide. Together, these advances usher in a new era of genomics research, enhancing the accuracy of genomic analysis, paving the path for precision medicine, and contributing to deeper insights into human biology.

## 1. INTRODUCTION

### 1.1. A Wondrous Map of Humankind

The sequencing of the human genome was a landmark achievement that revolutionized our understanding of human biology, enabling profound insights into the genetic basis of traits and diseases, as well as human evolutionary history (53, 55, 86). Before the availability of a human genome reference sequence, the field of human genetics relied heavily on family pedigree analyses, cytogenetic techniques, and low-resolution genetic assays to study the causes of genetic disorders (70). These early approaches were instrumental in laying the foundation for statistical methods such as association testing, fostering the concept of genetic counseling, and paving the way for more sophisticated molecular genetic studies. However, the lack of a human genome reference sequence limited the depth and breadth of insight—especially for complex traits that are influenced by numerous loci genome-wide.

Starting with a historic publication in 2001 (86), a human genome reference sequence has served multiple roles. One of the most important has been to act as a comprehensive catalog of genes and regulatory sequences that govern many aspects of human development, physiology, and pathology. Augmented by initiatives such as the Encyclopedia of DNA Elements (ENCODE) (46) and Roadmap Epigenomics (124) projects, the reference sequence has also enabled a deeper understanding of the complex networks of regulatory elements that orchestrate gene expression, shaping cellular functions and organismal development across healthy and disease states.

Relatedly, by facilitating family-based linkage analysis and association studies, the reference sequence exponentially accelerated the discovery and characterization of mutations, genes, and pathways that mediate variation in a vast array of traits and diseases. This progress is evidenced by the Online Mendelian Inheritance in Man (OMIM) database, which now catalogs the genetic basis of nearly 7,000 disorders and traits (9). Many common genetic risk factors have also been identified; as of October 2023, the National Human Genome Research Institute–European Bioinformatics Institute catalog of human genome-wide association studies (GWASs) (96) contained >500,000 associations reported in >6,500 published studies.

Another transformative aspect of the human genome reference sequence has been its role in the identification and analysis of genetic variation, which has propelled the fields of population genetics and comparative genomics (19, 80). At the most basic level, the reference sequence functions as an analytical resource and coordinate system that enables precise mapping of DNA sequence variation. The resulting information about genetic diversity within and between populations has fostered a deeper understanding of evolutionary processes such as historical migrations, changes in population size, and genetic adaptations to new environments.

**Figure 1:**
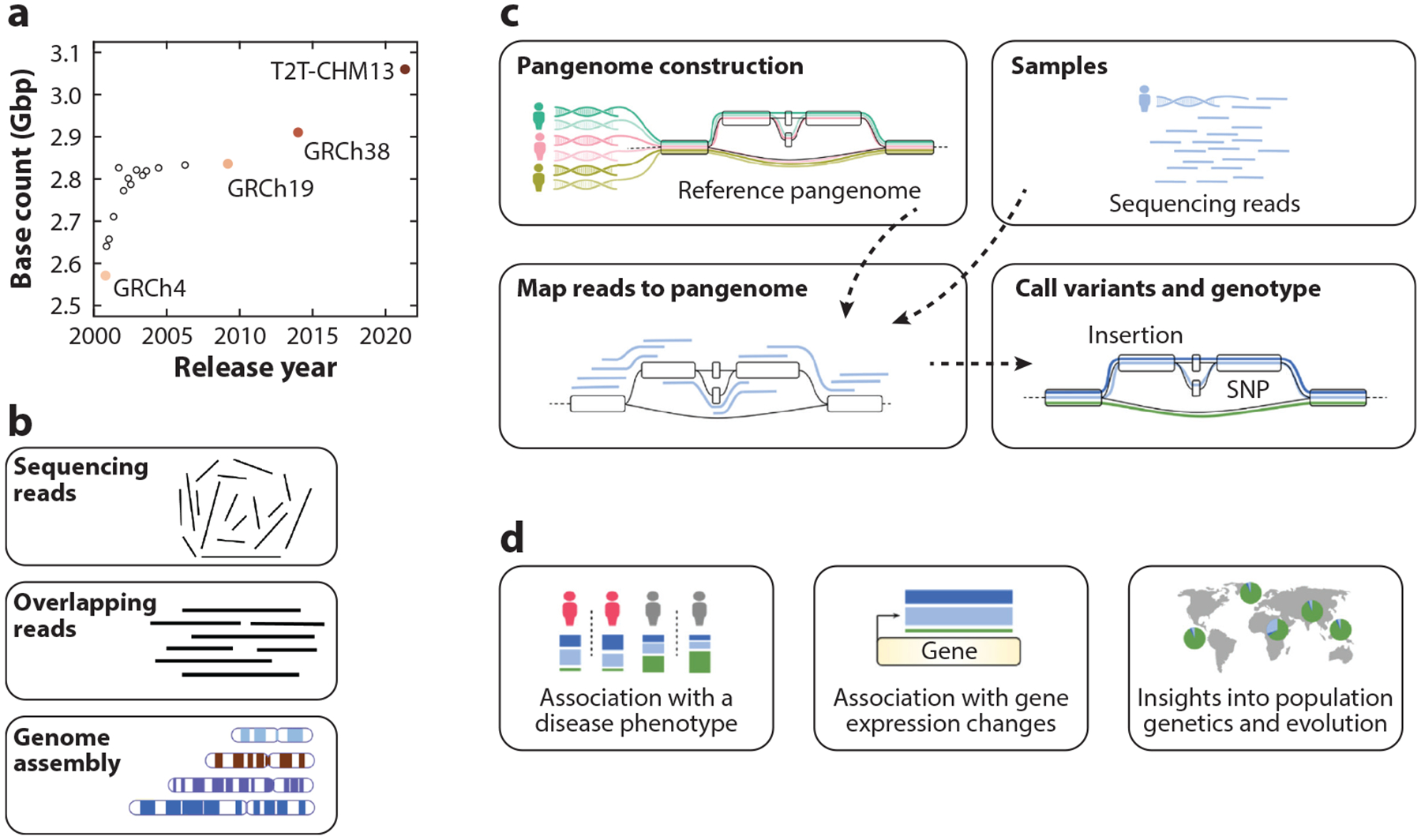
Overview of the process of human genome sequence assembly. (*a*) Sequence length improvements to the human genome reference sequence over time. (*b*) Overview of the genome sequence assembly process. First, individual sequencing reads are generated from a sample. Then, the sequencing reads are compared with each other to identify overlaps. Overlapping reads are then merged to generate a genome sequence. (*c*) Overview of pangenome reference assembly and analysis. First, the pangenome is assembled from multiple individual genome sequences, revealing commonalities and differences among them. Later, sequencing reads generated from other samples can be mapped (or aligned) to the pangenome reference to detect variants and establish genotypes. (*d*) Applications for human genome analysis. Abbreviations: SNP, single-nucleotide polymorphism; T2T, telomere-to-telomere. Panel *a* adapted from Reference 110; panels *c* and *d* adapted from Reference 133.

As we forge ahead, the human genome reference sequence will remain a cornerstone in the unfolding narrative of human genetics. However, the most widely used reference sequence (GRCh38) is not without limitations. Most immediately, large stretches, totaling nearly 200 Mbp (approximately the size of all of chromosome 3), remain unresolved and are represented by gaps or model sequences (manufactured sequences that match some features of the true sequences, such as their repeat content) (Figure 1a). It also contains numerous errors, especially involving misrepresentation of segmental duplications (SDs) and other complex regions of the human genome. More broadly, the use of a single linear reference sequence can introduce substantial reference biases where genetic differences between the reference and additional samples escape detection or are misreported.

This review focuses on two major advances that are addressing these issues. First, fully complete—or telomere-to-telomere (T2T)—human genome sequences have now been assembled, enabling more comprehensive analysis of complex and repetitive regions of the genome. Second, high-quality pangenomes, such as that produced by the Human Pangenome Reference Consortium (HPRC), offer a broader view of human genetic diversity. Major initiatives such as these are paving the way for groundbreaking discoveries and innovative strategies for understanding diseases and human biology.

### 1.2. Assembling the Original Human Genome Reference Sequence

After biologists first determined the structure of DNA in the 1950s, there was immediate interest in sequencing the human genome. However, decades of innovation were necessary to overcome the technical barriers that obstructed progress toward this goal. Formally launched in 1990, the Human Genome Project (HGP) was envisaged as a 15-year effort to achieve this herculean task through an international consortium involving the United States, the United Kingdom, France, Germany, Japan, and China (30). The fundamental challenge in sequencing the human genome, both then and now, is that no DNA-sequencing technology can sequence an entire human chromosome end-to-end. Consequently, human chromosome sequences need to be reconstructed from a large collection of individual DNA-sequencing reads in a computational process called de novo genome assembly.

Assembling a genome sequence is analogous to completing a jigsaw puzzle, in which individual DNA-sequencing reads are fit together to form larger segments (Figure 1b). Whereas jigsaw puzzle pieces fit together based on their physical shape and imagery, DNA-sequencing reads fit together based on shared nucleotide sequences. The core of this approach is to find pairs of reads where the end of one read matches the beginning of the next: a read overlap. Then, overlapping reads are aggregated with other overlapping reads to reconstruct larger and larger segments until a whole chromosome’s sequence has been assembled or, more commonly, until further extension becomes ambiguous. Like the most difficult parts of a jigsaw puzzle (e.g., a stretch of blue sky), the most difficult portions of a genome to assemble are repetitive sequences (e.g., those residing within centromeres, satellites, and transposable elements). Sequencing reads from such repetitive regions may fit equally well in multiple locations. While the earliest assembly approaches were based on relatively simplistic algorithms that did not guarantee optimal results, particularly in these complex regions (136), modern genome assembly algorithms rely on sophisticated graph-based data structures and algorithms (25, 84) to model and overcome all but the most challenging stretches of repetitive sequence.

By 1995, HGP researchers had created a detailed physical map of all human chromosomes, which divided and isolated the genome into more manageable parts that were cloned into bacterial cells, known as bacterial artificial chromosomes (BACs) (37). While BACs simplified the sequence assembly process, they ultimately suffered from the massive laboratory efforts required to construct and organize the large number of BACs needed to span the human genome. Around this same time, an alternate approach, called whole-genome shotgun sequencing, was developed that randomly sequenced different portions of an entire genome all at once, which were then assembled through a single large computation. Initially considered risky for a genome as large and complex as the human genome, a team led by Craig Venter and Hamilton Smith at the private company Celera Genomics demonstrated the potential feasibility of the approach through a series of increasingly larger genomes (4). Ultimately, both Venter’s team and the HGP adopted the shotgun method for finishing the outstanding portion of the human genome.

In a landmark announcement in 2001, the HGP and Celera Genomics jointly declared the completion of separate working draft sequences covering approximately 90% of the human genome (86, 140). These draft sequences provided a scaffold for identifying genes and regulatory elements. Two years later, the HGP released a much higher-quality (gold standard) human genome sequence, covering 99% of the euchromatic genome to an accuracy of 99.99% (77). This genome sequence (build 35) contained 2.85 billion nucleotides interrupted by only 341 gaps. The remaining euchromatic gaps were associated with SDs that the HGP researchers acknowledged would require substantial additional work and new methods to resolve.

Since the initial publications, the human genome reference sequence has undergone several refinements by the Genome Reference Consortium (GRC) to incorporate more comprehensive and accurate information. The most recent release, GRCh38, provides more complete coverage of the human genome, incorporating additional sequence data, especially in historically difficult-to-sequence regions (128). This includes better representation of centromeric regions, which are notoriously challenging to assemble due to their highly repetitive nature, through the use of model sequences. However, a truly complete human genome reference sequence remained elusive until 2022.

### 1.3. T2T-CHM13: A New Benchmark in Human Genomics

The Telomere-to-Telomere (T2T) Consortium was established in 2019 by Adam Phillippy and Karen Miga with the goal of developing a complete and accurate assembly of an entire human genome sequence (110). To achieve this goal, the T2T Consortium focused its initial efforts on the CHM13 cell line, which was derived from a complete hydatidiform mole. It possesses two nearly identical copies of the paternally inherited genome and is thus essentially homozygous genome-wide. This feature allowed the researchers to sidestep the challenge of distinguishing heterozygous from paralogous (i.e., duplicated) loci during the process of assembling a genome sequence from diploid cells.

The T2T sequence of the CHM13 genome (named T2T-CHM13) was enabled by several advancements in genome science, especially the advent of long-read DNA-sequencing technologies. The two most crucial technologies for this project were the availability of PacBio HiFi sequencing reads (145), which are extremely accurate (>99.9%) and span ~20 kbp in length, and Oxford Nanopore Technologies (ONT) ultralong sequencing reads (78), which have lower accuracy (~98%) but range from 100 kbp to >1 Mbp in length. The T2T Consortium developed sophisticated computational tools and sequence assembly algorithms that utilize these largely complementary data types. A key step was constructing the initial assembled sequences (contigs) from perfect overlaps between the HiFi sequencing reads, which resolved most repeats and assembled all but the most complex regions of the genome. Next, these contigs were resolved and merged together using the ultralong ONT sequencing reads.

Published in March 2022, T2T-CHM13 fills in all 200 Mbp of missing sequence from GRCh38, offering a more complete and accurate picture of the human genome, including the previously elusive centromeric regions, the short arms of all five acrocentric chromosomes, and other complex, repeat-rich loci (110) (Figure 1a). The new sequence also adds 1,956 gene predictions, 99 of which are predicted to be protein coding, and broadly improves the ability to resolve genetic variations and epigenetic activities across the genome. One year later, the T2T Consortium released the T2T-CHM13v2.0 genome sequence (123), which added a complete Y chromosome sequence from a separate donor. Completion of the T2T-CHM13v2.0 reference sequence marks a landmark achievement in human genomics and provides a complete sequence-based blueprint of all human chromosomes for the first time.

### 1.4. Capturing Human Genetic Diversity Within the Human Pangenome Reference

Although T2T-CHM13v2.0 represents a major achievement, no single genome sequence can represent the genetic diversity of the human species. Pangenome references offer a means to represent genetic diversity by integrating the genome sequences derived from multiple donors (Figure 1c). The term pangenome historically referred to the complete set of genes within a population or species (141), especially in prokaryotes (45). Because eukaryotic genomes are both far less gene dense and far less structurally labile than prokaryotic genomes, eukaryotic pangenomes generally aim to represent entire genomes and are not limited to transcribed regions (2).

Recent advancements in genome sequencing and assembly are now enabling production of population-scale genome sequences that can be used as input for developing pangenome references. Most notably, the HPRC has sequenced and assembled a set of globally diverse individual genomes to establish a draft human pangenome reference. While not yet true T2T sequences, these assemblies cover >99% of each genome and are >99% accurate at the structural and base-pair levels. Consequently, the HPRC pangenome adds 119 Mbp of euchromatic polymorphic sequences and 1,115 gene duplications relative to GRCh38. Approximately 90 Mbp of this additional sequence is derived from regions associated with structural variation.

Looking forward, we can expect a future human pangenome to form the basis of a common reference for the genetics and genomics communities (Figure 1d). It will almost certainly include many T2T genome sequences, with the goal of comprehensively and accurately capturing the large majority of common genetic variation that segregates across diverse human populations, thereby mitigating biases and facilitating biological discovery.

## 2. IMPLICATIONS OF THE FIRST COMPLETE HUMAN GENOME REFERENCE SEQUENCE

### 2.1. Value of a Complete Human Genome Reference Sequence

A reference genome is a central element of almost all clinical, comparative, and population genomic analyses. Using a reference genome, researchers can take advantage of the high sequence identity among human genomes (140) to make genetic studies more tractable. In a study involving multiple individuals, instead of assembling each person’s genome sequence de novo, it is generally more efficient to align sequencing reads to the reference sequence—with the expectation that >99% of bases will align perfectly—and noting mismatches as potential genetic variants. This approach is particularly appropriate when using short-read DNA-sequencing datasets (e.g., those using 100-bp Illumina sequencing reads). De novo sequence assembly approaches with such datasets would otherwise produce highly fragmented genome sequence assemblies (85). Beyond facilitating variant discovery and genotyping, a reference genome serves to standardize genomic analyses by providing a shared coordinate system by which to compare individuals’ genome sequences. This has direct implications for genomic medicine, as it allows for comparison of variant–trait or variant–disease associations across studies, regardless of study design.

**Figure 2:**
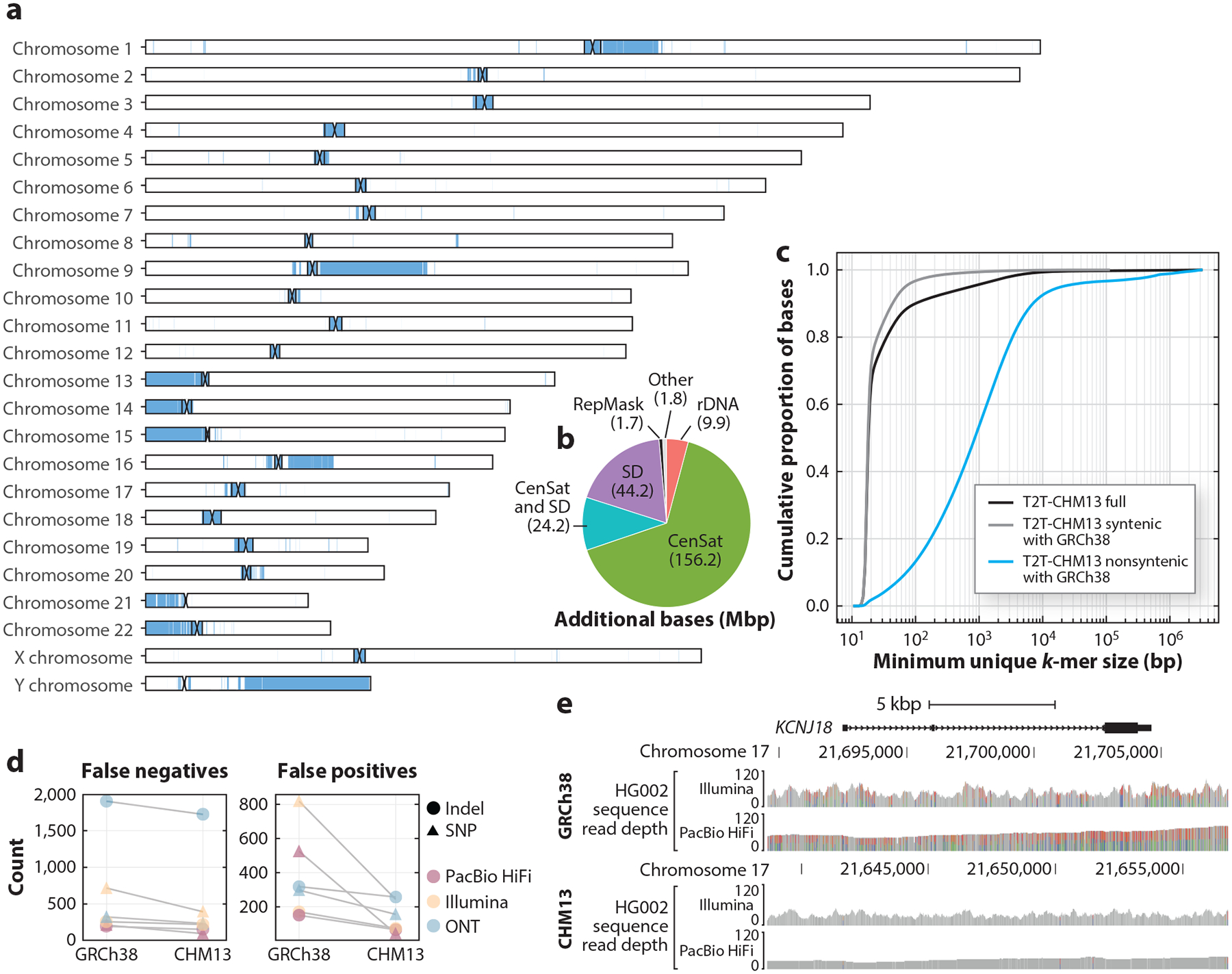
Overview of the first complete human genome assembly. (*a*) Ideogram of the T2T-CHM13v2.0 genome assembly. Regions of the assembly that are nonsyntenic with GRCh38 based on a whole-genome alignment between the two assemblies are shown in blue. (*b*) Breakdown of the sequence classes present in the regions of T2T-CHM13 that are nonsyntenic with GRCh38 (Y chromosome not included). (*c*) Mappability of the T2T-CHM13v2.0 genome based on minimum unique *k*-mer size, broken down by synteny with GRCh38. At each position in the genome, the minimum unique *k*-mer size is defined as the minimum number of bases (to the right) necessary to yield a unique sequence that does not appear elsewhere in the genome. Larger sizes imply poor mappability with short sequencing reads. (*d*) Performance of long- and short-read-based variant identification for a set of challenging medically relevant genes using T2T-CHM13 versus GRCh38. (*e*) Example of a medically relevant gene exhibiting improved mapping and variant identification using T2T-CHM13. *KCNJ18* falls within a collapsed duplicated region in GRCh38, which results in excessive read depth and spurious variants being identified; this is corrected using T2T-CHM13. Abbreviations: CenSat, centromeric satellite; indel, insertion or deletion; ONT, Oxford Nanopore Technologies; RepMask, RepeatMasker; SD, segmental duplication; SNP, single-nucleotide polymorphism; T2T, telomere-to-telomere. Panel *b* adapted from Reference 110; panels *d* and *e* adapted from Reference 5.

Because the human genome reference sequence is foundational for most human genetic analyses, the quality of that reference has broad implications for research across the field. Errors in the reference can propagate to downstream analyses, while gaps may preclude analyses of those genomic regions altogether. Here, we discuss the improvements of the T2T-CHM13v2.0 reference genome relative to the previous GRCh38 reference genome (Figure 2a,b) and its implications for studies of human genetic and epigenetic variation.

### 2.2. Improvements to Read Mapping and Variant Calling

Some of the most consequential improvements that a T2T reference affords pertain to mapping reads to the reference and calling genetic variants from those mappings. These improvements differ depending on the properties of the DNA-sequencing data (i.e., short-read versus long-read data). Accordingly, we summarize the effects on different DNA-sequencing technologies separately.

#### 2.2.1. Analyses of short-read data.

Currently, the most widely used technology for genome sequencing is short-read DNA sequencing (58). Consequently, it is crucial to understand how using a different reference genome alters our understanding of human genetic variation as analyzed using short reads. While the majority of sequence added in T2T-CHM13v2.0 relative to GRCh38 is in repetitive regions of the genome that complicate short-read alignments, T2T-CHM13v2.0 added >19 Mbp of sequence that is accessible for short-read analyses (meaning that short reads can be uniquely mapped to these regions) and made millions of additional revisions genome-wide (5, 123) (Figure 2c). The newly added sequence improves mapping and variant calling not only via the added sequence itself but also genome-wide, as it reduces the probability that sequencing reads derived from these regions will incorrectly map elsewhere.

Using short-read data from 3,202 individuals from 26 globally diverse populations sampled by the 1000 Genomes Project (1, 19), the T2T Consortium demonstrated that the use of the T2T-CHM13 reference broadly improves alignments. The researchers noted more mapped reads per sample (implying better representation of the full genome sequence), a higher proportion of properly paired read alignments (implying greater structural consistency), and a lower average mismatch rate for aligned reads (implying fewer spurious differences). These advantages extend to variant calling, where there were fewer variants called per sample (reflecting the correction of errors in the reference) but more variants called across samples (implying the discovery of previously hidden variation, especially within newly resolved regions). Notably, for T2T-CHM13v1.0, the newly added sequence relative to GRCh38 overlaps >200 protein-coding genes and includes tens of thousands of high-confidence variants per sample. These include variants in proximity to known GWAS candidate regions (18) and pathogenic variants (87), suggesting potential functional, biomedical, and evolutionary relevance and motivating future research.

#### 2.2.2. Analyses of long-read data.

Given the repetitive nature of much of the newly added regions in T2T-CHM13, its greatest benefits will be realized through the use of long-read DNA-sequencing technologies (151). Specifically, T2T-CHM13v2.0 added >82 Mbp of unique sequence that is newly alignable using either PacBio HiFi or ONT ultralong sequencing reads (measured by uniqueness of 1-kbp segments) (Figure 2c). More broadly, the T2T Consortium demonstrated that T2T-CHM13 substantially improves alignment and variant calling using long sequencing reads. In an analysis of 17 long-read datasets, the authors observed lower per-read mismatch rates and more uniform coverage across the genome (5). Within the newly added regions of the genome in T2T-CHM13v1.0, HiFi and ONT reads allowed for the discovery of >1 million single-nucleotide variants (SNVs) per sample, only 4–5% of which were able to be identified using short reads. Long reads are also valuable for discovering larger structural variants (SVs), and the authors identified >22,000 SVs overlapping the newly added regions, highlighting the value of this technology when combined with T2T assemblies.

### 2.3. Reduction of Spurious Variants

Beyond adding ~200 Mbp of sequence missing from the GRCh38 reference, the T2T-CHM13 reference also corrects ~30 Mbp of problematic sequence in the GRCh38 reference. One of the most prevalent forms of error in the GRCh38 reference has been the incorrect representation of SDs—long stretches of nearly identical sequence that occur multiple times throughout the genome (142). When copies of the SD are falsely collapsed in the reference sequence, it can lead to the false identification of an abundance of spurious heterozygous variants. This is caused by the small differences among the duplicated sequences, which are incorrectly reported within the falsely collapsed regions as variants for which all individuals are heterozygous (25). Collapsed duplications and other missing sequences can also lead to a bias toward identifying insertions (21), a pattern that was observed in GRCh38 and corrected in T2T-CHM13 (5, 123). GRCh38 also contains numerous insertions and deletions (indels) and SNVs that are private to the reference but absent from the 1000 Genomes Project samples (5). While any given sample is expected to carry some private variation, the magnitude of private variation in GRCh38 is highly suggestive of technical error (5).

These errors in the reference hinder the accurate identification of variants across hundreds of protein-coding genes, including many genes of medical relevance. Using a benchmark set of identified high-confidence variants, one study found that the use of T2T-CHM13v1.0 reduced the rate of identifying false-positive and false-negative variants, broadly improving genetic analysis involving a panel of 273 clinically relevant but technically challenging genes (143) (Figure 2d,e).

### 2.4. Enhancing the Accuracy and Completeness of Functional Genomic Analyses

In addition to the advantages in detecting genomic variation, T2T references promise improvement for the analyses of data coming from myriad sequencing-based functional genomic assays. Such data facilitate gaining a greater understanding of regulatory processes, including epigenomics, transcription, and translation, which collectively mediate cellular functions. Most such analyses involve aligning sequencing reads to a reference. As such, the robustness of these assays strongly depends on the accuracy and completeness of the reference, as well as any necessary auxiliary annotations (e.g., gene annotations for RNA-sequencing analysis).

The T2T-CHM13 reference genome unlocks new opportunities to investigate function within structurally complex regions (54, 108). While historically excluded from analyses, these loci are known to harbor strong associations with numerous clinical phenotypes and to exhibit evidence of historical natural selection (149). Initial functional studies that relied on the T2T-CHM13 reference also demonstrated that investigating these complex regions will require the use of long-read sequencing to overcome the limitations of short-read sequencing methods (5). One area where long reads may be particularly useful is in the discovery and characterization of human splice isoforms. While several recent long-read RNA-sequencing studies have expanded the catalog of human transcriptional diversity, they have not yet leveraged the use of T2T-CHM13, as gene and other annotations remain less mature compared with GRCh38 (57, 122, 148). Moreover, the extent to which these (typically low-abundance) novel transcripts are translated and contribute to phenotypic diversity remains an important open question. Similar questions emerge from long-read epigenetic studies, which enable measurement of diverse features of methylation, chromatin accessibility, and other features. These assays are also greatly enhanced by long-read analyses with the T2T-CHM13 reference genome (54).

## 3. FROM ONE TELOMERE-TO-TELOMERE GENOME TO MANY

The first T2T human genome sequence was the result of a massive collaborative effort across several institutions and substantial manual effort, but this accomplishment paved the way for more cost-effective and automated assemblies of additional genome sequences for humans and other species. Here, we review recent lessons and progress toward generating automated T2T sequence assemblies.

### 3.1. Sequence Data Requirements

As was used for generating T2T-CHM13, automated gapless assembly algorithms use a combination of ONT ultralong sequencing reads and PacBio HiFi sequencing reads, but they also add other DNA sequence data to enable long-range phasing. Such phasing is important to resolve biological variations between maternal and paternal alleles and also eliminates a major source of sequence assembly errors. To provide long-range phasing, proximity ligation DNA sequencing, such as short-read Hi-C, can be used to link alleles deriving from the same haplotype as well as scaffold sequence contigs over long spans. Alternatively, phasing can be achieved by sequencing the genomes of the parents in a parent–child trio, which provides markers in the sequences that can be used for phasing. Currently, the recommendation for attaining the highest-quality automated assembly of a human genome sequence requires >60× coverage with HiFi reads, >30× coverage with ONT ultralong reads (100 kbp or longer), and either Hi-C or parental short reads (121).

Alternative data can also assist in T2T sequence assembly and phasing. Optical mapping, which labels DNA molecules at specific motifs and then images these molecules, is available using technologies from companies such as Bionano. Changes in the distance between labels or the pattern of the labels are used to order and orient sequence scaffolds or identify SVs. Another frequently used method is single-cell DNA template strand sequencing, in which one DNA strand in each cell is selectively labeled; this produces a directional short-read library that is useful for phasing and correcting inversion errors (114).

### 3.2. Telomere-to-Telomere Assembly Algorithms

A few sequence assembly algorithms have been developed to generate T2T or nearly T2T sequence assemblies, given sufficient data volume and quality. The Verkko assembler was developed from the lessons learned in generating T2T-CHM13 (121). Briefly, the algorithm begins by building a de Bruijn graph from the long, accurate sequence reads. A de Bruijn graph identifies overlaps between the reads by decomposing them into shorter fixed-length substrings called *k*-mers. These *k*-mers form the nodes in the graph, and edges are added between consecutive *k*-mers across all sequencing reads. Consequently, overlaps are identified through nodes (*k*-mers) shared by multiple reads. The de Bruijn graph from Verkko utilizes the MBG (Minimizer-Based Sparse de Bruijn Graph) tool to create a memory-efficient graph with an initial default *k*-mer size of 1,001. GraphAligner (120) is then used to align the ultralong sequencing reads to the constructed graph, and these alignments are used to resolve repeats and fill in gaps. Substantial coverage of both ultralong sequencing reads and highly accurate sequencing reads is required to generate a T2T sequence. For example, a draft T2T sequence of the human HG002 sample with Verkko required 105× PacBio HiFi and 85× ONT ultralong-read coverage (121).

Similarly, recent versions of hifiasm can integrate ultralong-read data to generate T2T sequences (25). This method has reportedly generated a T2T human genome sequence in one day as well as T2T sequences of polyploid plant genomes. This algorithm was initially designed for use with PacBio HiFi sequencing reads (26) but is now able to use ultralong sequencing reads (hifiasm-UL). There are a few notable differences between Verkko and hifiasm-UL in their ultra-long read integration. Notably, hifiasm-UL relies on string graphs rather than de Bruijn graphs for the sequence assembly and builds different string graphs for each data type (long, accurate reads and ultralong reads) before merging the two graphs together. Additionally, hifiasm can also integrate Hi-C data into the hybrid sequence assembly process.

### 3.3. Early Examples and Future Work

The recent assembly of the human Y chromosome sequence showcases several methodological advances in the generation of genome sequences. The initial T2T-CHM13v1.0 sequence excluded the human Y chromosome because the CHM13 cell line does not possess a Y chromosome. Thus, a substantial effort was made after the initial T2T-CHM13 release to generate the first T2T Y chromosome sequence (123). Much of the Y chromosome is repetitive; GRCh38 includes only half of the chromosome’s >62 Mbp of sequence. Methodologically, the generation of the T2T Y chromosome sequence built on the strategies used to derive the original T2T-CHM13 sequence, with a few changes to accommodate the unique nature of human sex chromosomes. In this case, tangles between the string graphs of the X and Y chromosomes, particularly at pseudoautosomal regions, were automatically resolved using ONT sequencing reads. This strategy yielded >30 Mbp of new sequence and uncovered 41 potential protein-coding genes. Published in tandem with the T2T Y chromosome sequence were 43 near-T2T Y chromosome sequences assembled using Verkko (66).

In parallel to sequence assembly efforts, technical advances are also enabling improvements to base-pair sequence accuracy. This correction process, termed polishing, typically requires the integration of additional highly accurate data types. For example, polishing of T2T-CHM13 improved the average base quality from Q70.2 to Q73.9 (i.e., from an average of 1 error every 10.5 Mbp to 1 error every 24.5 Mbp) (100). The remaining errors were enriched in regions with low HiFi sequencing-read coverage (~0.3% of the assembly). Addressing these remaining challenges, the T2T Consortium is actively working toward a completely gap-free and error-free (i.e., Q100) assembly of the HG002 genome sequence using refined algorithms and the newest DNA-sequencing technologies (101). T2T assemblies created using these approaches are in development for dozens of additional humans in the next few years.

These T2T sequence assembly strategies are applicable beyond human DNA samples. The T2T Consortium is also actively working to generate T2T genome sequences of nonhuman primates, including gorilla, chimpanzee, bonobo, orangutan, and siamang. The initial sex chromosome sequences were released in December 2022 as part of phase I of this project (102), and the autosome sequences are expected in 2024. Furthermore, a near-complete *Arabidopsis* genome sequence, which utilized many of the validation strategies developed by the T2T Consortium, resolved centromeric regions that had been absent from previous genome sequences (107). More broadly, we anticipate the generation of T2T genome sequences for all of the most important plant, animal, fungal, and microbial species within the next decade.

## 4. ADVANCING TOWARD A COMPLETE GENOMIC PICTURE

### 4.1. The Elements of a Human Pangenome

The availability of high-quality genome sequences for many human and nonhuman samples has motivated the recent focus on pangenomes to capture and understand genetic diversity. There is an increasing recognition that the core of a eukaryotic pangenome should be based on a collection of reference-quality genome sequences (144). However, additional components are necessary to make the pangenome a useful resource. Accordingly, we define a pangenome based on three “A” components, without regard to specific formats or data structures that may constitute them:

- Assemblies: A collection of haplotype-resolved T2T or near-T2T reference genome sequence assemblies. For human, this could encompass existing reference sequences like GRCh38 (128), T2T-CHM13 (110), and earlier versions like GRCh37 (28). The inclusion of previous reference sequences facilitates backward compatibility within the pangenome coordinate system.
- Alignments: An alignment of the haplotypes, serving to delineate homology relationships between different parts of the genome. By incorporating existing reference sequences, this alignment facilitates referencing variations using the established coordinates.
- Annotations: Functional annotations for each sequence, with essential annotations rendered in the coordinates of respective reference sequences. The alignment inherently represents homology relationships, facilitating mutual consistency of gene annotations.

To work with these three A’s, we can conceive of multiple potential representations. To date, graph-based representations have received the greatest attention. However, differing representations do not change the key components of the pangenome, only how they are accessed and utilized. For example, alternate methods decompose the pangenome into sets of variable blocks (17, 97) or form alignments implicitly by representing all matches among a collection of linear sequences (125).

### 4.2. Surveying Human Variation

Any two human genomes differ by multiple classes of variation. To create a complete human pangenome, it is necessary to understand and model all these types, a summary of which is given in Table 1.

The most common types of variations are SNVs and short indels. They greatly outnumber other types of DNA sequence variants, with an average of 4 million SNVs and 1 million short indels per human genome relative to GRCh38 (19). SVs are less frequent, although they represent a particularly important class because they involve larger differences in alleles. This blanket category encompasses larger indels as well as duplications, translocations, and inversions as well as other events at least 50 bp in size (71). Studies have identified tens of megabases of DNA within common polymorphic SVs (32, 43, 93). The average human genome contains 25,000–35,000 SVs (93). This estimate has increased with the ability to determine more of the genome, particularly through the use of long-read DNA-sequencing technologies. However, SVs in heterochromatic sequences are still missing from most current analyses. These include high-copy-number tandem duplications, such as satellite arrays and the 45S rDNA arrays on the acrocentric chromosome short arms (63, 74, 110). Earlier cytogenetic studies showed that the satellite arrays that constitute the entirety of human centromeres can vary by >1 Mbp in length (8, 103, 104). With multiple forthcoming T2T or near-T2T sequences, it should finally be possible to more confidently assemble the full complement of satellite variation. However, alignment of such highly repetitive sequences is an active research challenge (20), and it remains difficult to discern individual variations.

### 4.3. Draft Human Pangenomes

Building on the long history of the International HapMap Project and other major efforts cataloging human variation (76), several initiatives have sought to augment the reference sequence to better reflect population genetic diversity (130). Using data from 910 DNA samples from the Consortium on Asthma Among African-Ancestry Populations in the Americas (CAAPA) cohort, Sherman et al. (130) identified nearly 300 Mbp of human genome sequence that was absent from GRCh38. More recently, the Human Genome Structural Variation Consortium (HGSVC) (43) produced and compared de novo genome sequences of individuals of diverse ancestry.

**Figure 3:**
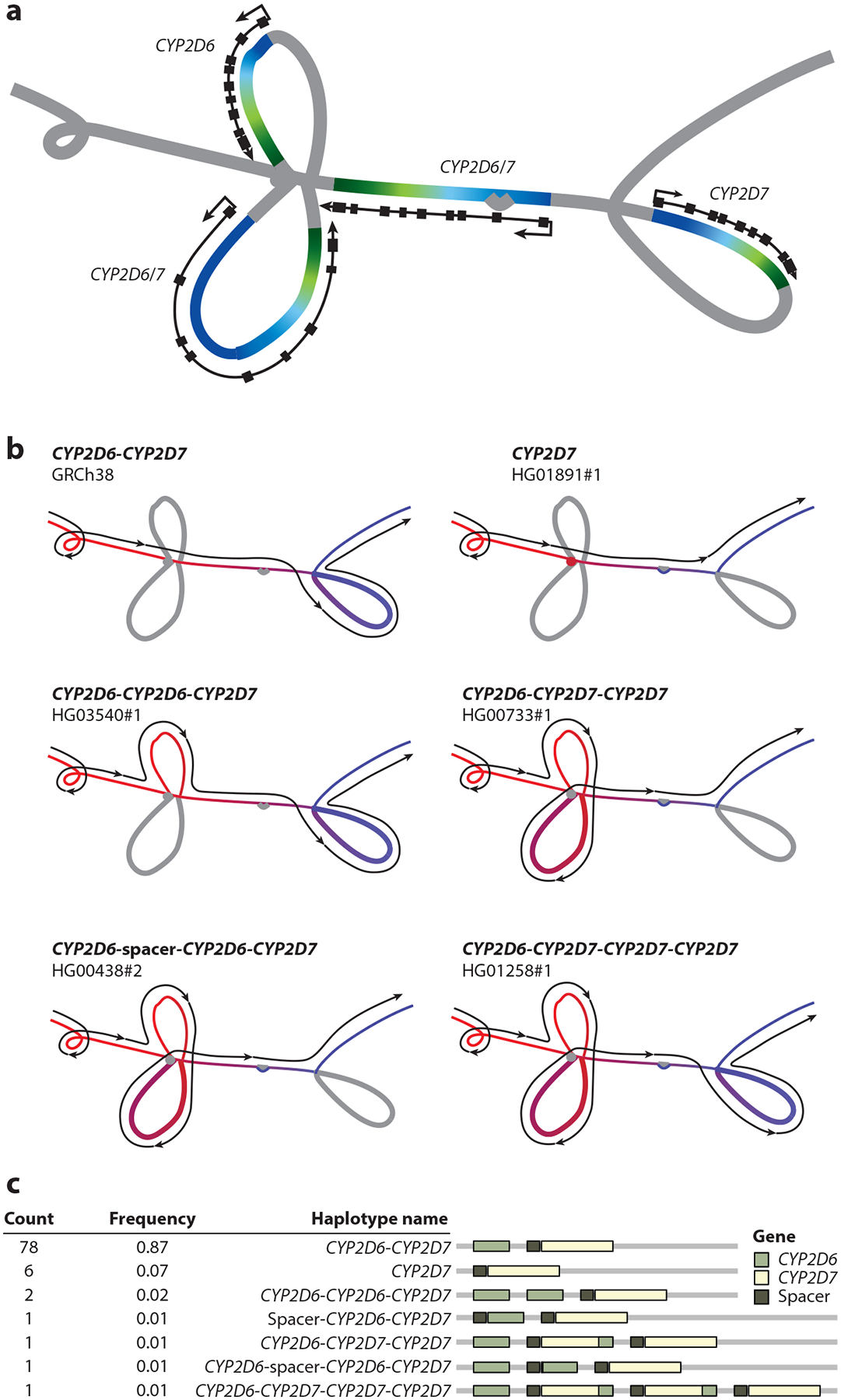
Illustrating the HPRC pangenome with an example. (*a*) The structural haplotypes of the *CYP2D6* and *CYP2D7* genes called from the Minigraph-Cactus HPRC pangenome graph. The color gradients are based on the relative positions of the genes: Green represents the head of a gene, and blue represents the end of a gene. (*b*) Different paths taken by different structural haplotypes in the graph. The color gradient is based on path position: Red represents the head of a path, and blue represents the end of a path. (*c*) Frequency and linear structural visualization of all structural haplotypes called by the Minigraph-Cactus graph. Abbreviation: HPRC, Human Pangenome Reference Consortium. Figure adapted from Reference 93 (CC BY 4.0) with assistance from Shuangjia Lu.

Building on these efforts, the HPRC (93) released a complete pangenome reference with assemblies, alignments, and annotations that encompass 47 DNA samples selected from the 1000 Genomes Project (1). Each sequence is comparable in quality to the existing reference sequence, and relative to GRCh38, they collectively add >120 Mbp of polymorphic euchromatic sequence, 90 Mbp of which is derived from larger SVs. The addition of new heterochromatic sequence is considerably larger than this, but it is difficult to quantify given the limitations of current algorithms in aligning this highly repetitive sequence. The sequences include >1,200 gene copy-number polymorphisms and an additional 0.5–6 Mbp of genic sequence per sequence relative to GRCh38. An example of this genic complexity is shown in Figure 3, which illustrates the *CYP2D6/7* locus in the HPRC pangenome.

Additional human population pangenome projects are also emerging. For example, the Chinese Pangenome Consortium used similar methods to the HPRC in generating a pangenome of 58 individuals from 36 Chinese ethnic groups (50). The Global Alliance for Genomics and Health recently announced an international Human Pangenome Project to attempt to coordinate efforts, with the goal of fostering standards, interoperability, and ultimately integration. Such efforts will be crucial to realizing the synergies among the complementary pangenomes that have already occurred and those that are sure to come.

### 4.4. Human Pangenome Growth and Sample Selection

In the future, the human pangenome created by the HPRC will include the genome sequences of at least 350 individuals (i.e., 700 haplotypes) that reflect global genetic diversity (144), with the goal of representing all common variants (defined as those occurring at a 1% or higher frequency in the global population). To that end, the project has identified three core criteria for sample selection: to select samples that contribute or maximize the number of new common variants, to select samples that maximize genetic dissimilarity, and to target known underrepresented populations.

To optimize the first two criteria, the HPRC has developed an algorithm built on earlier work (79) that utilizes preexisting sets of identified variants derived from short-read exome- or genome-sequencing projects. This approach maximizes coverage of common variants without relying on categorical population labels that introduce errors by discretizing patterns of variation that actually vary continuously. Given the elevated rates of heterozygosity observed in individuals of African ancestries, this algorithm exhibits a pronounced tendency to select these genomes. Further, given the known underrepresentation of people from regions like western Asia, northern Africa, and Oceania in genomic databases, the HPRC algorithm aims to specifically bolster diversity from these areas.

While the first two criteria rely solely on genetic data, the third criterion emphasizes community engagement and informed consent for participants from underrepresented groups. This is particularly important in light of the failings of some past studies (11, 34, 40, 41, 106). In recognition of this fact, the HPRC integrates considerations of both scientific goals and ethical, legal, and social implications into its strategy for recruiting diverse participants. These efforts help guide development of informed-consent documents and agreements, strategies for communicating with participants (12, 138, 139), and participant privacy (10, 59). Such considerations are essential for all pangenome development projects moving forward, as researchers should seek to represent greater diversity within the pangenome while avoiding breaches of privacy, scientific racism, eugenic interpretations of research, and other potential harms.

### 4.5. Pangenome Construction

The task of constructing a pangenome from a set of sequence assemblies poses a substantial technical challenge. As in the assembly of individual genome sequences, errors may occur during the inference process due to statistical uncertainty or limitations in the algorithms. Unlike genome sequence assembly, which has broadly recognized quality metrics (e.g., contiguity, completeness, and correctness), there is no single accepted criterion for pangenome construction that can evaluate the inference. Pangenomes may differ in the set of alignments and annotations they include without any being incorrect. Moreover, the best choice of criterion may vary by application.

It is instructive to consider the strategies used by the leading methods to demonstrate the range of principles that guide pangenome construction. The program Minigraph attempts to retain consistency with a preexisting reference sequence by prohibiting graph motifs that disrupt the linearity of the reference. Moreover, it keeps the graph structure simple by intentionally omitting small variants (89). The Minigraph-Cactus method extends Minigraph to achieve full base resolution, including small variants, using multiple sequence alignment heuristics around base-level breakpoints (68). The Pangenome Graph Builder method eschews structural simplicity as an explicit goal and instead structures its graph using alignments that indicate any homology relationship (51). Several methods represent the pangenome as a colored de Bruijn graph, which discards most positional information in the assemblies in favor of efficient algorithms and data structures (6, 73). Finally, the Pangenome Research Toolkit constructs variably coarse-grained local graphs of specified genomic regions, primarily as a means to infer and display repeat structure over a set of linear haplotypes within the region (27).

These approaches share many challenges in common. Chief among these is identifying the alignments that should comprise the pangenome, which proves especially challenging for large-scale repeats, such as variable number tandem repeats (VNTRs), SDs, and tandem repeat arrays. Similarly, pangenome annotations (e.g., for transcripts) pose challenges in light of haplotype, cell type, and tissue diversity. High-quality annotations typically require extensive manual curation and experimental characterization in multiple conditions (46, 49). It is impractical to repeat this process for every sequence used in generating a large pangenome. In some cases, existing annotations can be projected from the reference sequence onto haplotypes with computational methods (48, 131), but there are inevitably cases where this process fails. This strategy also has limited ability to discover functional annotations of sequences that are present across the pangenome but missing from the existing reference sequences.

## 5. THE POWER OF PANGENOMICS: BROADER APPLICATIONS IN GENOMIC STUDIES

### 5.1. Mitigating Reference Biases in Genomic Analyses

**Figure 4:**
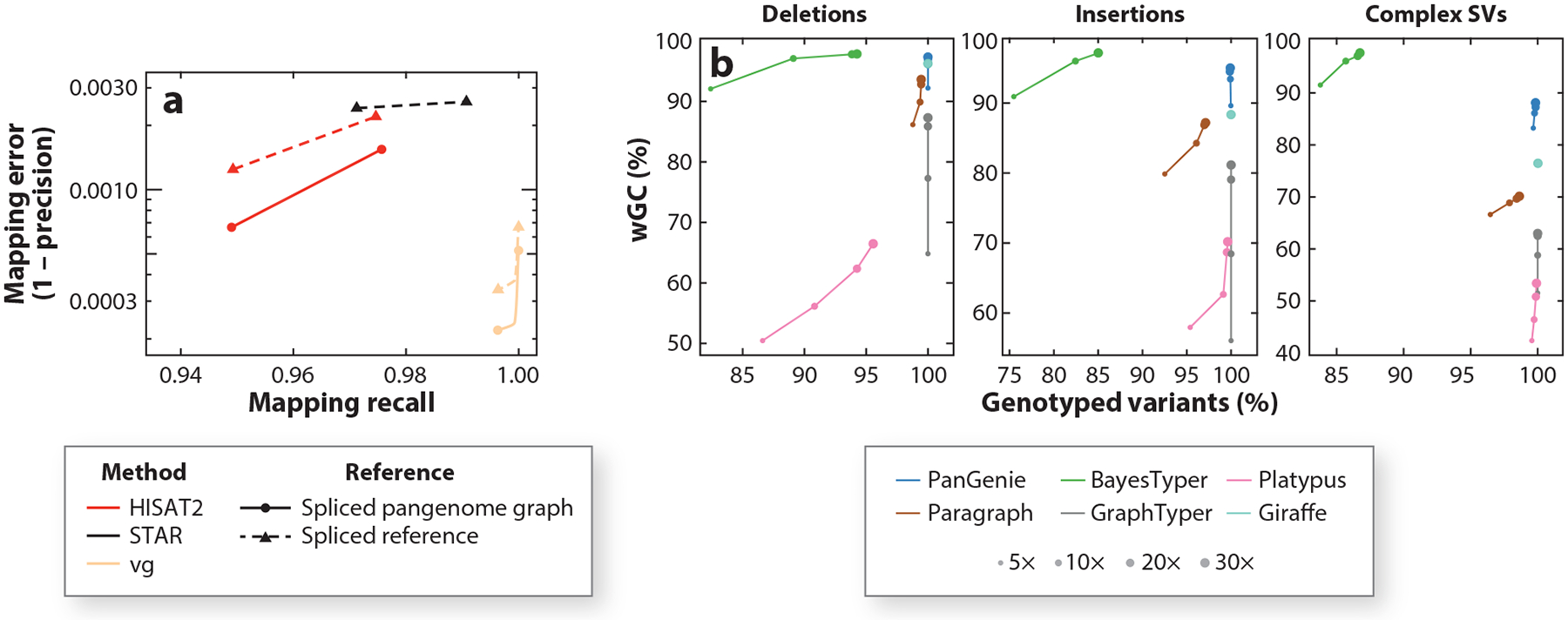
Broader applications of pangenomes. (*a*) Mapping simulated RNA-sequencing reads to a spliced reference (*dashed lines*) or spliced pangenome (*solid lines*). STAR takes only known splicing information into account, while HISAT2 and the vg toolkit also further integrate genetic variants, which results in substantially fewer incorrectly mapped sequencing reads. (*b*) Genotyping SVs from the HGSVC catalog using different pangenome-based approaches. This panel shows wGC values in nonrepetitive regions, at different coverages (point size), for sample NA12878, which was removed from the catalog for a leave-one-out evaluation. Complex SVs are all variant sites that are not biallelic deletions or insertions. PanGenie is able to genotype the vast majority of SVs accurately. Abbreviations: HGSVC, Human Genome Structural Variation Consortium; HISAT2, Hierarchical Indexing for Spliced Alignment of Transcripts 2; STAR, Spliced Transcripts Alignment to a Reference; SV, structural variant; vg, variation graphs; wGC, weighted genotype concordance. Panel *a* adapted from Reference 132; panel *b* adapted from Reference 44 (CC BY 4.0).

Pangenome-based approaches mitigate reference bias when mapping sequencing reads and identifying variants by incorporating known genetic variation (45). The benefit is most pronounced in regions where alignment is challenging, such as those harboring SVs or high genetic diversity, including hundreds of genes of clinical relevance (143). The reduction in bias also naturally benefits the quantitative analysis of functional sequencing data, such as that produced by chromatin immunoprecipitation followed by sequencing (ChIP-seq) (61) or RNA sequencing (132). Pan-transcriptomes that represent both variants and known splicing events map sequencing reads from RNA sequencing more accurately to quantify transcript expression levels (132) (Figure 4a). Mapping reads to the pangenome also improves the performance of existing variant-identifying methods. For example, the HPRC used pangenome read-mapping methods to reduce errors in identifying small variants genome-wide by 34% compared with conventional approaches, with even greater improvement among complex, clinically relevant loci (93). Similarly, parental pangenomes have been used to boost the performance of short-variant identification in an undiagnosed rare-disease cohort (98).

Pangenome-based approaches are also useful to resolve complex situations such as nested or overlapping forms of genetic variation (88) as well as SVs and tandem repeats (45). Underscoring this point, pangenome-based methods implemented in tools such as PanGenie (44), Paragraph (24, 149), GraphTyper (13), and the vg toolkit (133) have allowed for the efficient analysis of long-read-discovered SVs in short-read sequencing data from thousands of samples. These applications have revealed allele frequencies (133) and evolutionary signatures (149) for many novel SVs as well as enabling the inclusion of these SVs in GWASs (13). Haplotype information may further improve pangenome-based genotyping (Figure 4b), as demonstrated by methods such as PanGenie, in which loci are genotyped based on both their own read support and that of nearby variants in linkage disequilibrium (44). Along these same lines, pangenomes provide better allelic representations for repeat-rich variants such as tandem repeats (42). For example, using the latest human pangenomes from the HGSVC and the HPRC, Lu et al. (95) introduced a de Bruijn graph–based approach to better estimate the length of VNTRs across samples and detect differences in their sequence composition.

### 5.2. Insights into Population Genetics and Evolution

Patterns of variation among genome sequences derived from contemporary human populations provide a record of historical evolutionary events, including changes in population size, population divergence, gene flow, and adaptation to new environments (109). While most historical analyses have focused on abundant classes of short variation, pangenome approaches promise greater understanding of larger and more complex forms of structural variation that may play an outsize role in evolution (129).

Several pangenome-based studies have quantified the marginal increase in the cumulative amount of unique sequence or number of SVs with each additional sample, noting that these asymptotic curves are well approximated by power-law distributions and saturate at different rates in different human populations (118, 130). Notably, human populations are strongly enriched for rare variation as a consequence of recent population growth (81), which in turn implies that much rare structural variation remains to be discovered in pangenomes with larger and larger numbers of sequences—especially those from the diverse populations within Africa that remain poorly represented in human studies (112).

It is worth noting that most of the African genome sequences in the CAAPA pangenome (130) align with high completeness and sequence identity to T2T-CHM13 (5), which is derived from a sample of largely European ancestries, in contrast to the predominantly African American ancestry of GRCh38. This counterintuitive observation suggests that the absence of these sequences from GRCh38 was less a product of its ancestry composition and more a product of the technical challenges of an incomplete reference sequence. Indeed, as a consequence of human demographic history, common variation tends to be shared across human populations, originating in the ancestral human population within Africa prior to migrations across the globe (14). The few exceptions include loci such as *LCT*/*MCM6*, *FADS1*/*FADS2*, and *SLC24A5* that likely conferred a fitness advantage in specific environments and swept to high frequencies in subsets of populations (47). Meanwhile, rare variation tends to exhibit greater geographic stratification simply due to its more recent origins. As such, while the use of population-specific linear reference sequences (38) is well intended for reducing the effects of reference bias (16, 35), its utility may remain limited for rare variation. This further motivates pangenome approaches that seek to capture haplotype variation, agnostic of sample ancestry, as well as ever-larger pangenomes (52).

### 5.3. Enhancing Disease Association Studies

**Figure 5:**
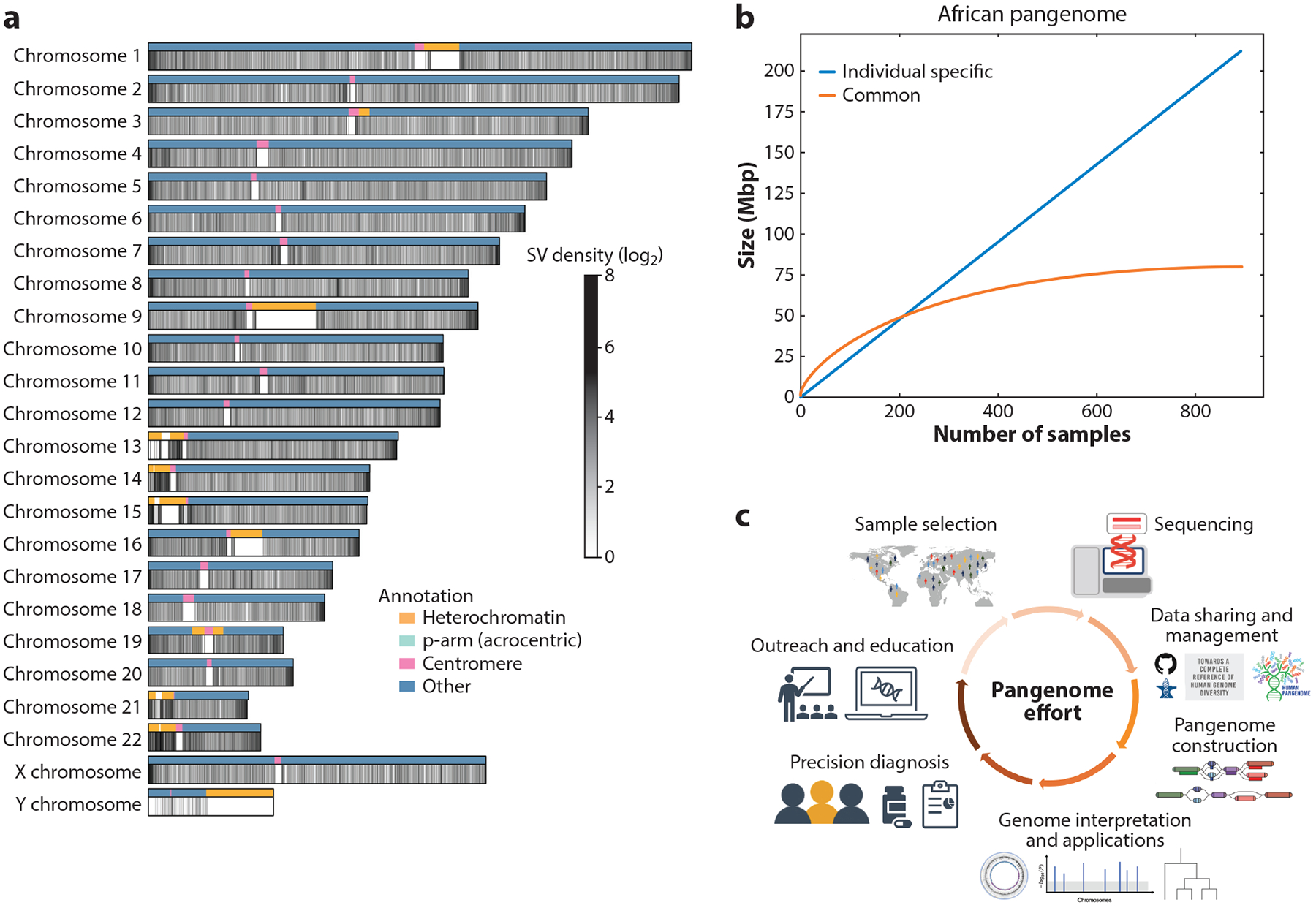
Opportunities and needs for pangenome research. (*a*) Density of SVs (≥50 bp) across T2T-CHM13. The variants are sourced from the HPRC-MC VCF file (93). Colored bands indicate genomic annotations for T2T-CHM13. (*b*) Growth of common and individual-specific sequences within the 910 individuals of the African pangenome population (130), where common sequences are defined as sequences present in at least two samples included in the pangenome. The orange line represents the average sizes of the common sequences in a certain number of individuals after randomly sampling 1,000 times. The blue line represents the average sizes of individual-specific sequences from the same samples. (*c*) Overview of pangenome efforts. Abbreviations: HPRC, Human Pangenome Reference Consortium; MC, Minigraph-Cactus; SV, structural variant; T2T, telomere-to-telomere; VCF, Variant Call Format. Pangenome construction illustration adapted from Reference 105 (CC BY 4.0); right-hand outreach and education illustration provided by Darryl Leja/National Human Genome Research Institute (public domain).

Despite affecting many crucial genes and gene families, complex structural changes are broadly missed in current genetic and genomic studies based on GRCh38 (111). The shift toward graph-based pangenomes improves the performance of variant identification, contributing to building a more complete variant landscape (Figure 5a). Knowledge of these variants contributes to a comprehensive understanding of complex, medically important genes (92). For example, lipoprotein(a) is an essential factor in coronary heart disease risk, and its expression level correlates with the *LPA* gene length. However, it is difficult to accurately determine the copy number of kringle IV type 2 (*KIV-2*) in *LPA* because the repeat structure frustrates short-read mapping when using GRCh38. The HPRC identified new *KIV-2* variants and constructed a comprehensive diversity map of the *LPA* gene, demonstrating the potential of pangenome graph approaches in deciphering complex and clinically significant regions of the human genome. It also reported that genes that vary in copy number accounted for 0.6–4.4 Mbp of additional genic sequences per haplotype; this includes genes that are critical to human health, such as the amylase, β-defensin, and *NOTCH2NLC*–*NOTCH2NLB* genes. Recently, the Chinese Pangenome Consortium discovered 1,575 assembly-specific sequences spanning 72.41 Mbp that are absent in the HPRC pangenome (50). These sequences affect >1,200 genes involved in key metabolic processes and are associated with several major diseases, including cancer, schizophrenia, and nervous system disorders. These endeavors show the diversity within the human genome and elucidate the intricate interplay between genetic variants and pathologies, offering valuable insights for future genomic and clinical research.

Several studies have integrated pangenome-based methods into GWASs (79, 90). Traditional GWASs utilize only single-nucleotide polymorphisms as markers, which has contributed to the challenge of missing heritability (3). In recent years, GWASs have increasingly adopted two additional markers generated from pangenome-based methods, SVs and *k*-mers, which has fostered novel discoveries and enhanced the accuracy of estimated associations (65). Researchers have also constructed pangenome graphs to analyze complex diseases characterized by an intricate nexus of genetic and environmental factors. Hokin et al. (72) utilized a pangenome graph to associate complex diseases, including schizophrenia, with certain genotypes and enhanced the performance of risk prediction. In addition to identifying new associations, pangenomes will likely advance fine-tuning of established GWAS-identified candidate regions and thereby localize causal variants driving these signals (33).

Relatedly, the application of pangenomes has extended to the mapping of genetic variations associated with gene expression—often referred to as expression quantitative trait loci (eQTLs)—and their influence on downstream phenotypes (3). In 2021, Sirén et al. (133) used pangenome-based methods to identify and genotype SVs in 5,202 samples, resulting in the discovery of eQTLs related to diverse processes such as immunity and neuronal cell excitability. Additionally, ongoing efforts aim to investigate the impact of VNTRs on gene expression. Lu et al. (94) constructed a repeat-pangenome graph to capture the repeat structural and sequence diversity of VNTR loci, discovering 346 expression-associated VNTRs, of which 344 (99.4%) were previously undiscovered, which provided new clues about disease risks. For example, the expression-associated VNTRs were identified in *ERAP2*, which has implications for ankylosing spondylitis and Crohn’s disease, and in *KANSL1*, which is associated with Koolen–de Vries syndrome and Parkinson’s disease.

### 5.4. Applications and Unique Challenges in Nonhuman Species

The reference-bias problem, inherent in a single linear reference sequence, is not limited to human studies. On top of the existing challenges present in assembling genome sequences of nonmodel organisms, constructing a pangenome for nonhuman species can be complicated by features such as greater genetic diversity, larger genomes, more repetitive sequences, and polyploidy (56). The strawberry, for example, possesses a highly complex allo-octoploid genome that originated from four separate diploid progenitors, while mistletoe possesses a haploid genome size of ~90 Gbp, 30 times the size of the human genome.

Despite these challenges, pangenome-based approaches have proven valuable for studying genomes across the tree of life, especially for plants of agricultural importance (36), where they can guide agronomic trait selection. For example, a tomato pangenome resolved >200,000 SVs, demonstrated SV genotype-to-phenotype relationships, and ultimately led to crop improvement (7). Meanwhile, a rice pangenome allowed the discovery of SVs targeted by local adaptation to diverse environments, a topic of increasing importance in light of climate change; specifically, the authors identified SVs within the pangenome that confer blast pathogen resistance (117).

The evolutionary and comparative biology fields have also been quick to utilize pangenomes to study broad evolutionary questions (150). Bozan et al. (15) explored speciation in potato relatives through a pangenome-based presence–absence variation analysis, providing insights on the role of transposable elements in speciation events. A pangenome study of the strawberry clade focused on resolving the phylogenetic relationships between diploid *Fragaria* species; the pangenome revealed a new species, and the authors presented the pangenome as a model for evolutionary genomics (116). Additional high-quality genome sequences and pangenomes from even more diverse clades could potentially reveal additional speciation events and selective sweeps, providing valuable insight into the evolutionary forces that shape genomes.

## 6. FUTURE OPPORTUNITIES FOR PANGENOME RESEARCH

### 6.1. Challenges for Pangenome Use and Adoption

While tools for pangenome analyses have matured, several obstacles have hindered further adoption. One of the most pressing challenges is the need to develop new computational tools tailored to facilitate pangenome storage, manipulation, and visualization, as well as the downstream use of pangenomes for alignment, variant discovery, genotyping, and other applications.

The maturation of computational pangenome-based studies has been undergirded by a growing suite of computational tools to store and manipulate pangenomes. The field has increasingly settled on the Graphical Fragment Assembly (GFA) format as an easily parsed, interpretable interchange format for sequence graphs. More efficient formats are also available for the assemblies, with or without the accompanying alignment (39, 134). Many graph manipulations and analyses are implemented in toolkits like vg (52), odgi (64), and gfatools (89). However, the space of manipulations that can be performed on pangenomes is much wider than for linear reference sequences, especially in light of the widespread structural variation present in human genomes (Figure 5a). Many analyses are therefore based on ad hoc scripts, given the significant holes in the software tool infrastructure.

Early methods for read alignment to pangenomes were time and memory intensive, and they often failed to outperform methods for the linear reference sequence (133). To address these challenges, modern graph-based aligners such as HISAT2 (83) and Giraffe use efficient computational indexes to store and query the pangenome (133). Such pangenome-based analyses typically output results in conventional linear reference sequence-based formats, such as Binary Alignment Map (BAM) and Variant Call Format (VCF). The projection back to linear reference sequences can result in information loss that partially defeats the purpose of the pangenome. While GFA is increasingly adopted as the standard for pangenome sequence-read mapping, there is no corresponding graph-based alternative to VCF, which limits the advantages of pangenome graphs (68).

Relatedly, most current methods for constructing pangenome graphs do not explicitly consider the evolutionary relationships among the sequenced genomes. Instead, all paths through the pangenome are considered equally likely a priori. This simple approach is expedient but biologically naive. In reality, genomes are related by a series of correlated phylogenies across consecutive genomic intervals, which vary due to recombination (91). These relationships are described by the ancestral recombination graph (ARG)—a data structure that comprehensively encodes genealogical relationships across the genome based on coalescence and recombination events (60, 75). Recent years have seen advances in the ability to efficiently infer, store, and analyze ARGs for large samples, facilitating inference of evolutionary and demographic events (119, 135). To date, pangenome graphs and ARGs have largely developed in parallel, but future pangenome research may draw inspiration from ARGs. For example, knowledge of evolutionary relationships among the samples that make up a pangenome may improve analysis of additional samples. An example is recent work that leverages knowledge of allele frequencies and haplotype structure to enhance genotyping accuracy (22). Evolutionary relationships may be especially beneficial for resolving paths through complex regions of a pangenome graph, as certain paths may be more likely than others based on sample relationships inferred from the rest of the genome.

### 6.2. Scaling to Large Samples from Diverse Populations

There are several ongoing efforts to build population-specific (50, 92) and global human pangenome reference sequences (93). While varying in scale, these typically include genomic data from a few hundred individuals, though ongoing efforts aim for nearly 1,000 individuals. Larger samples, in turn, facilitate the integration of more rare and population-specific variation into the pangenome. This can be seen in the distinct growth patterns among common and individual-specific variants present in existing large pangenomes (92, 130). These efforts show that while common variants can be identified quickly, the amount of individual-specific sequence continues to grow with pangenome size (Figure 5b), suggesting that the total pangenome size is unbounded. The addition of more samples will only help to build a more comprehensive picture.

Given their scale and standardization, data from sources such as the UK Biobank (67), Trans-Omics for Precision Medicine (TOPMed) (137), and the *All of Us* Research Program (126) may offer valuable sources of data for pangenome construction involving genome sequences from hundreds of thousands of individuals. They also present a mounting technical challenge. Computational analyses such as multiple-genome alignment become challenging at this scale. To overcome this issue, recent studies have instead relied on sequential pairwise or progressive alignments that cannot always guarantee accuracy. Even the optimal order of sequential alignment remains an open question (82).

It is important to note that while pangenomes offer a step toward greater representation of human genetic diversity, they are still limited to the variation present among the individuals from which they are constructed. Accordingly, those responsible for generating the underlying human genome sequences must be aware of potential biases in sample collection (69), including socioeconomic factors and barriers to participation. Once data are collected, there need to be streamlined means of collaborating on and sharing these data, as well as combining data from multiple studies (146). Cloud computing can be an effective tool to achieve these goals (127). Doing so can open the door for more data to be included in each effort and make it easier to incorporate data from a range of sources, as long as consistent data and metadata standards are used (23).

### 6.3. Translating Research to Clinical Applications

Precision medicine has gained prominence in healthcare, with the goal of providing tailored diagnoses and therapeutic strategies based on each patient’s genomic information (31). Because precision medicine relies on knowledge of associations between genetic variation and clinically relevant phenotypes, its value depends on the data on which these associations are based. Unfortunately, previous GWASs have been strongly biased toward individuals of European ancestries, to the exclusion of other global populations (112). This may limit the relevance of results to other populations due to (*a*) differences among populations in the patterns of linkage disequilibrium between a tag variant and the unknown causal variant and (*b*) the inability to discover causal variants that are rare or absent in the population sample (99, 147).

Efforts to generate large, globally diverse resources that address these biases are underway, including TOPMed and the *All of Us* Research Program. They seek to generate genome sequences and phenotypic data from a diverse cohort of hundreds of thousands to millions of individuals (126, 137). Integrating these datasets with pangenome reference sequences generated from diverse samples will improve genotyping of complex variation (some of which may be rare or population specific) and thereby also facilitate discovery of clinically relevant variation. A recent study (62) successfully applied a pangenome-based approach to discover candidate causal SVs for several rare diseases using 668 haploid genomes from the Genomic Answers for Kids program (29) and the HPRC. While the cohort used in this study was much smaller and less diverse than those of either TOPMed or the *All of Us* Research Program, it highlights the potential advantages of integrating large, diverse cohorts with pangenome-based approaches. Such comprehensive analyses would improve social equity in precision medicine by uncovering the sources of variation in disease susceptibility in diverse cohorts, which enables clinicians and researchers to optimize the efficacy of corresponding treatments.

Presently, the pangenome is evolving through multifaceted collaborations, gathering global interdisciplinary working groups committed to varied sample collection, genome sequencing, and the optimization of pangenome construction. These collaborative efforts not only promote a deeper understanding of biology but also underscore the potential of pangenomes in precision medicine. We expect strengthened collaboration, continuous resource enhancement, broader sharing, and education initiatives to further advance pangenome development (Figure 5c).

## Figures and Tables

**Table 1:** Summary of variants in the euchromatic portion of a human genome

Variant type	Average number of sites (thousands)^a^	Average sum of variant length (Mbp)^b^	Percentage of diploid genome^c^
All	5,045.39	44.24	0.763
SNV (including MNPs)	3,992.73	3.99	0.069
Indel	1,021.73	3.63	0.063
SV^d^	30.93	36.62	0.631
STR	2.65	0.19	0.003
VNTR	12.58	1.36	0.023
Other low complexity	2.58	0.13	0.002
SD	0.55	6.25	0.108
Mobile element	6.18	1.91	0.033
LINE1	0.98	0.91	0.016
ERV	0.64	0.27	0.005
Alu	3.49	0.48	0.008
SVA	1.07	0.25	0.004
Inversion	0.15	23.2	0.400
Unclassified/mixed	6.23	3.58	0.062

Abbreviations: ERV, endogenous retrovirus; indel, insertion or deletion; LINE1, long interspersed element 1; MNP, multiple-nucleotide polymorphism; SD, segmental duplication; SINE, short interspersed element; SNV, single-nucleotide variant; STR, short tandem repeat; SV, structural variant; SVA, SINE-VNTR-Alu; VCF, Variant Call Format; VNTR, variable number tandem repeat.

a The average number of sites observed of a given variant type within each genome.

b The average total length of variant sites.

c The percentage of a diploid genome that each variant type represents, assuming a 5.8-Gb diploid euchromatic genome length. The values exclude heterochromatin due to uncertainty around assembly and alignment for all variants except inversions, where estimates are from Porubsky et al. (115) and not necessarily restricted to euchromatic sequence.

d SVs include all structural variants; the remaining rows are SV subclasses. Unclassified/mixed denotes a class of SVs for which reliable annotation could not be given. SV counts, excluding inversions, were calculated from Minigraph (89) VCF files released as part of a paper by Liao et al. (93), provided by Heng Li and Wen-Wei Liao. Small-variant numbers are also from Liao et al. (93) and were calculated using PacBio HiFi sequencing data and DeepVariant (113).
